# Supplementary material for: AAV-ie enables safe and efficient gene transfer to inner ear cells
Source: Nat Commun. 2019 Aug 19;10:3733. doi: 10.1038/s41467-019-11687-8 (PMC6700137; doi:10.1038/s41467-019-11687-8)
Supplement: Supplementary file 2 — Supplementary Information [file 41467_2019_11687_MOESM2_ESM.pdf]

AAV-ie enables safe and efficient gene transfer to inner ear cells

Fangzhi Tan[1,14*], Cenfeng Chu[1,2,3,14], Jieyu Qi[4,5,14], Wenyan Li[6,14], Dan You[6], Ke Li[3,7,8], Xin Chen[2,3], Weidong Zhao[6], Cheng Cheng[9,10], Xiaoyi Liu[1,2,3], Yunbo Qiao[11], Bing Su[1,2,3], Shuijin He[2], Chao Zhong[7], Huawei Li[6*], Renjie Chai[4,5,6,12,13,*], Guisheng Zhong[1,2,5*]

Corresponding author e-mails: Fangzhi Tan (tanfzh@shanghaitech.edu.cn), Huawei Li (lihuawei63@gmail.com), Renjie Chai (renjiec@seu.edu.cn) or Guisheng Zhong (zhongsh@shanghaitech.edu.cn)

Supplementary Information

(Contains Supplementary Figures 1-8 with Legends)

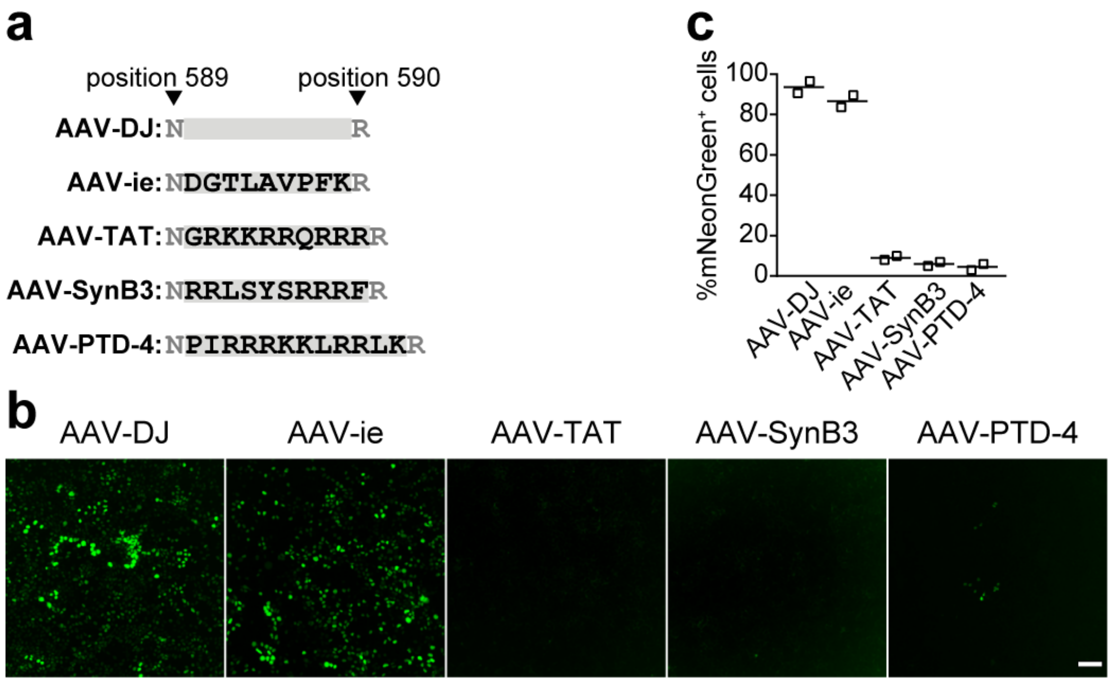

Supplementary Figure 1 Analysis of the *in vitro* transduction efficient of AAV-DJ and its variant vectors. (**a**) Several CPPs and peptide (DGTLAVPFK) were inserted into AAV-DJ's VP1 capsid at the position between N589 and R590. (**b**) HEK 293T cells were infected with AAV vectors at a MOI of 1000, and 48 h later, NLS-mNeonGreen expression was observed by fluorescent microscope and NLS-mNeonGreen positive efficiencies (**c**) of these AAV vectors were shown. Data are shown as Mean±SEM. Scale bar, 50 µm. Source data are provided as a Source Data file.

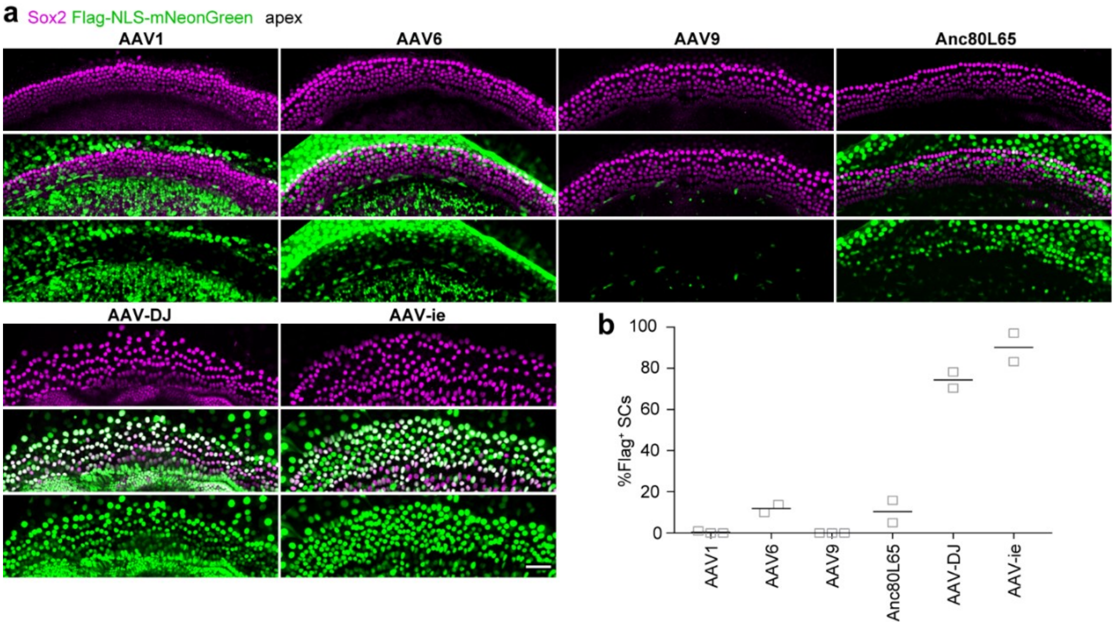

Supplementary Figure 2 Transduction of organotypic explants of murine cochlea with AAV serotypes. (**a**) Representative confocal images of an *in vitro* comparison of several AAV serotypes for NLS-mNeonGreen transgene expression in cochlear explants of C57BL/6 mice. Images show the expression at the cochlear apex for all serotypes after incubation with 2×10^{10} GC particles for 60 h. Green: Flag-NLS-mNeonGreen, magenta: Sox2. Scale bar, 50 µm. (**b**) Percentage of Flag-NLS-mNeonGreen positive SCs per 100 µm after 60 h incubation. The horizontal bars represent mean value. N=2-3 mice. Source data are provided as a Source Data file.

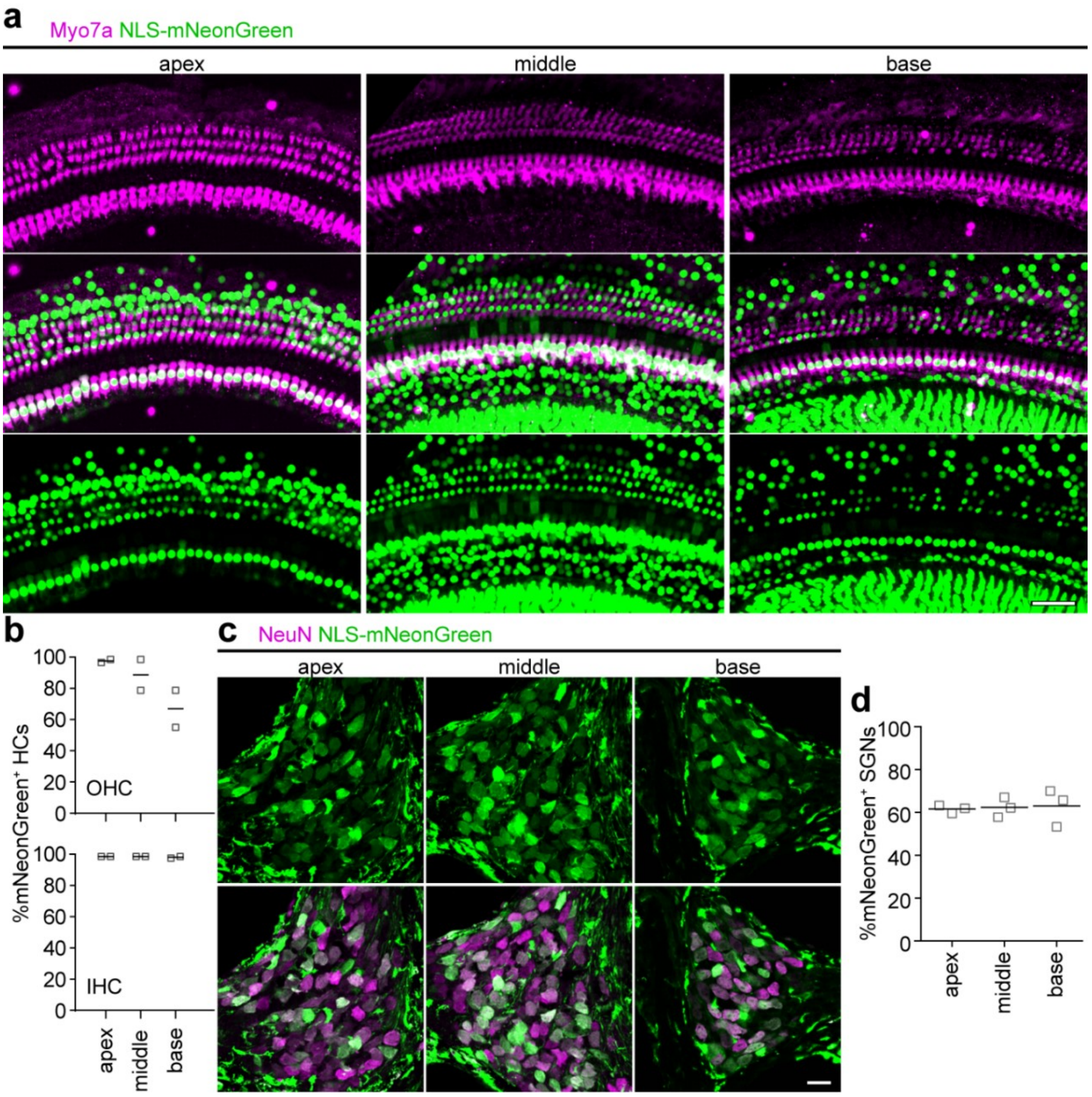

Supplementary Figure 3 *In vivo* transduction of AAV-ie in hair cells and spiral ganglion neurons. (**a**) Representative confocal images of apical, middle and basal regions injected with AAV-ie-NLS-mNeonGreen at the dose of 6.9×10^9 GC particles per ear. Green: NLS-mNeonGreen, magenta: Myo7a. Scale bar, 50 µm. **(b)** Quantification of inner and outer hair cell infection efficiency. Horizontal bars represent mean value. N=2 mice. Source data are provided as a Source Data file. **(c)** Representative confocal images of SGNs infected with AAV-ie-NLS-mNeonGreen at the dose of 6.9×10^9 GC particles per ear. Temporal bone was harvested at day 27 after microinjection with 1.5 µL of AAV stock solution at P3. Green: NLS-mNeonGreen, magenta: NeuN (a marker for spiral ganglion neurons). Scale bar, 20 µm. **(d)** Percentage of NLS-mNeonGreen positive SGNs. Horizontal bars represent mean value. N=3 mice. Source data are provided as a Source Data file.

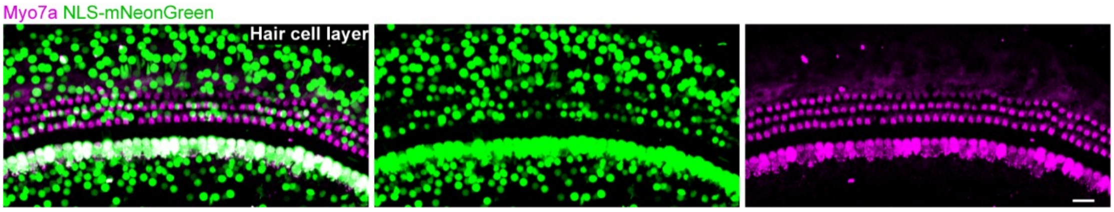

Supplementary Figure 4 *In vivo* transduction of AAV-ie in adult cochlear hair cells. Representative confocal images of cochlear hair cells. Green: NLS-mNeonGreen, magenta: Myo7a. Scale bar, 20 μm. Mice were injected at P30 with AAV-ie-NLS-mNeonGreen at dose of 1×10^{10} GCs per ear and the tissue was harvested at P45.

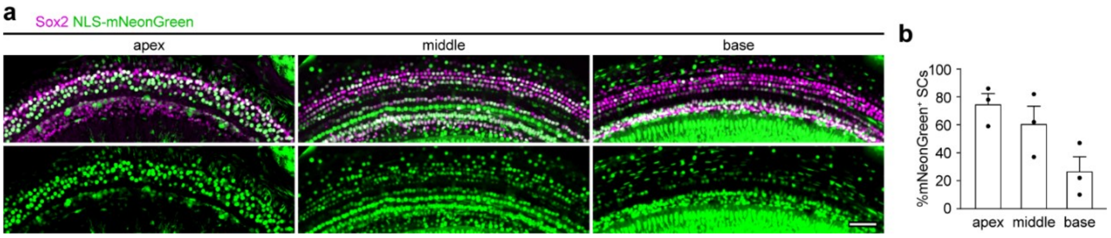

Supplementary Figure 5 *In vivo* transduction of low-dose AAV-ie in neonatal mice cochleas. (**a**) Images of the apical, middle and basal regions injected at P3 with AAV-ie-NLS-mNeonGreen at the dose of 3.6×10^{9} GCs per ear. The cochlea was harvested at P17 and stained with anti-Sox2 antibody (magenta) and imaged for mNeonGreen (green). Scale bar, 50 µm. (**b**) Percentage of NLS-mNeonGreen positive SCs per 100 µm corresponding to (a). Data are shown as Mean±SEM. N=3 mice. Source data are provided as a Source Data file.

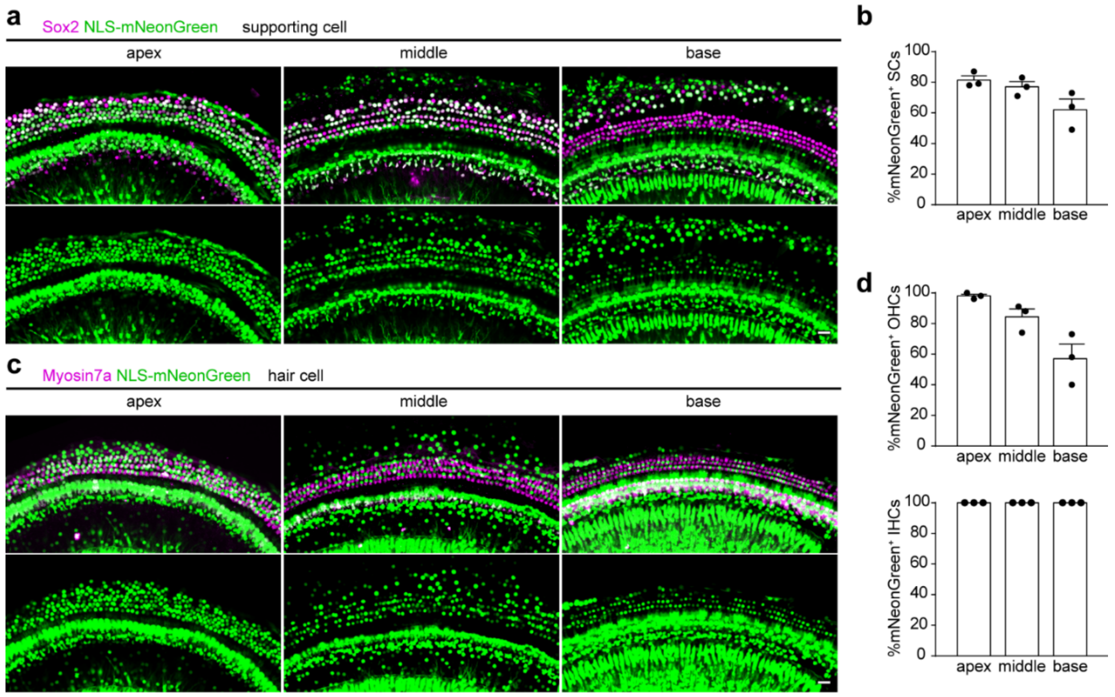

Supplementary Figure 6 Long-term *in vivo* cochlear transgene expression mediated by AAV-ie. Mouse cochleae from P3 mice injected with AAV-ie-NLS-mNeonGreen (1×10^{10} GCs per ear). The tissues were harvested at P30 fixed and stained with anti-Sox2 antibody (**a**, magenta) or anti-Myo7a antibody (**c**, magenta) and imaged for mNeonGreen fluorescence (green). Scale bar, 20 μm. (**b**) Percentage of NLS-mNeonGreen positive SCs per 100 μm corresponding to (a). Data are shown as Mean±SEM. N=3 mice. Source data are provided as a Source Data file. (**d**) Percentage of NLS-mNeonGreen positive HCs per 100 μm corresponding to (c). Data are shown as Mean±SEM. N=3 mice. Source data are provided as a Source Data file.

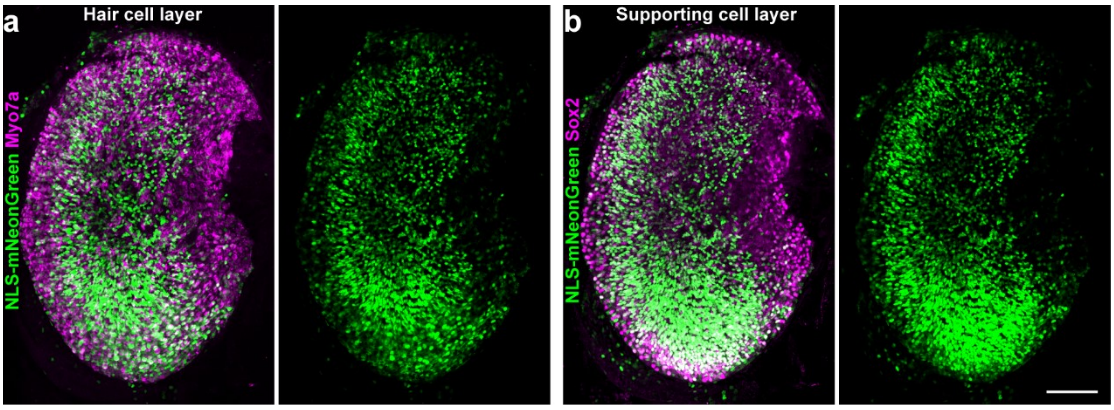

Supplementary Figure 7 *In vivo* transduction of AAV-ie in adult utricule. Representative confocal images of hair cells **(a)** and supporting cells **(b)** in utricular epithelia. Scale bar, 100 µm. Mice were injected at P30 with AAV-ie-NLS-mNeonGreen at dose of 1×10^{10} GCs per ear and the tissue was harvested at P45.

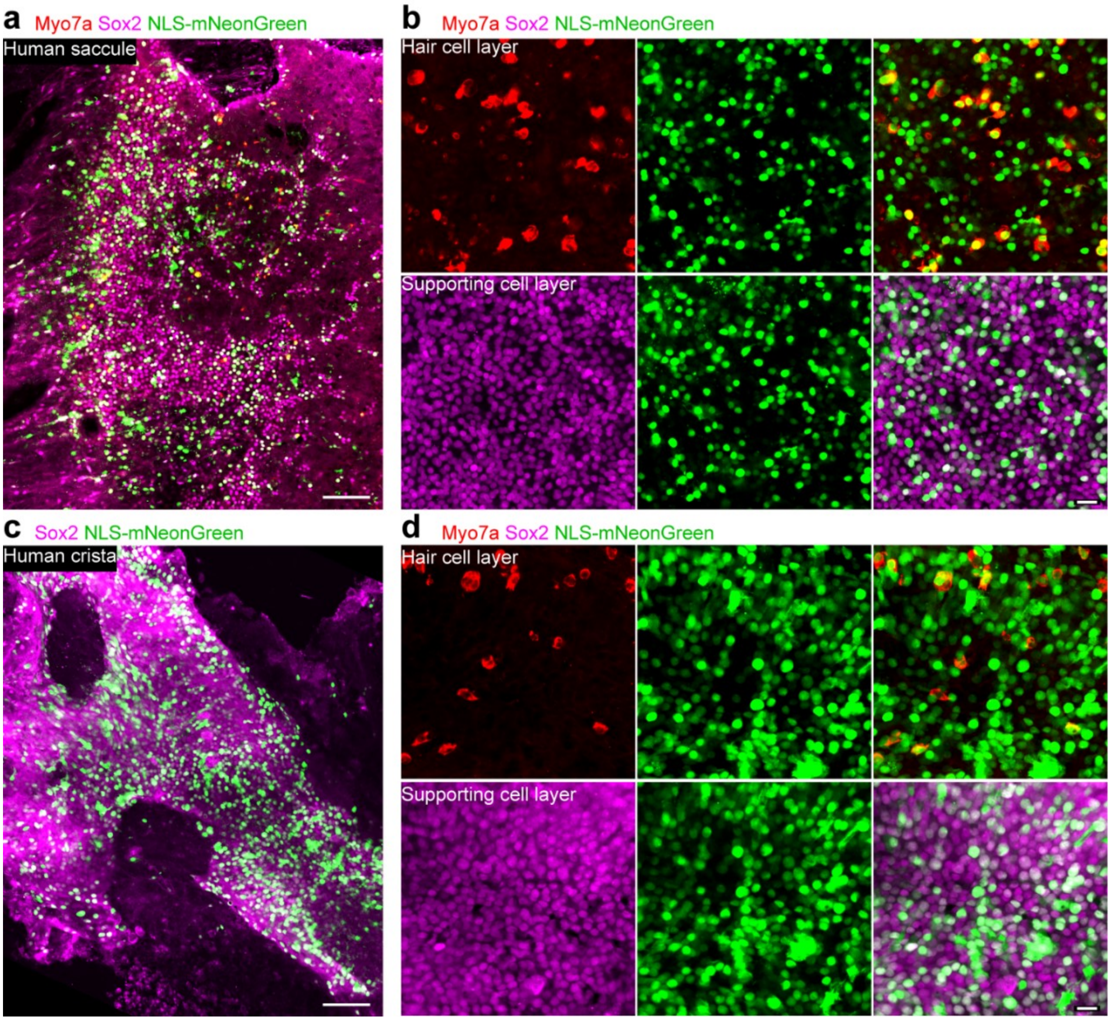

Supplementary Figure 8 *In vitro* transduction of AAV-ie in human saccule and crista. The human tissues were exposed to 5×10^{10} GCs AAV-ie-NLS-mNeonGreen for 24 h, cultured for 7 d, fixed, stained with anti-Myo7a antibody (red) or anti-Sox2 antibody (magenta) and imaged for mNeonGreen fluorescence (green). **(a)** The sensory epithelium of an adult human saccule. Scale bar, 100 µm. **(b)** Magnified regions from (**a**) in HC layer and SC layer. Scale bar, 20 µm. (**c**) The sensory epithelium of a crista of semicircular canal. Scale bar, 100 µm. **(d)** Magnified regions from (**c**) in HC layer and SC layer. Scale bar, 20 µm.
