# Supplementary material for: AAV-ie enables safe and efficient gene transfer to inner ear cells
Source: Nat Commun. 2019 Aug 19;10:3733. doi: 10.1038/s41467-019-11687-8 (PMC6700137; doi:10.1038/s41467-019-11687-8)
Supplement: Supplementary file 3 — Reporting Summary [file 41467_2019_11687_MOESM3_ESM.pdf]

nature research

Corresponding author(s): Guisheng Zhong, Renjie Chai, Huawei Li, Fangzhi Tan

Last updated by author(s): Jul 15, 2019

Reporting Summary

Nature Research wishes to improve the reproducibility of the work that we publish. This form provides structure for consistency and transparency in reporting. For further information on Nature Research policies, see Authors & Referees and the Editorial Policy Checklist.

Statistics

For all statistical analyses, confirm that the following items are present in the figure legend, table legend, main text, or Methods section.

n/a	Confirmed	
☐	☒	The exact sample size (*n*) for each experimental group/condition, given as a discrete number and unit of measurement
☐	☒	A statement on whether measurements were taken from distinct samples or whether the same sample was measured repeatedly
☐	☒	The statistical test(s) used AND whether they are one- or two-sided<br>*Only common tests should be described solely by name; describe more complex techniques in the Methods section.*
☒	☐	A description of all covariates tested
☒	☐	A description of any assumptions or corrections, such as tests of normality and adjustment for multiple comparisons
☐	☒	A full description of the statistical parameters including central tendency (e.g. means) or other basic estimates (e.g. regression coefficient) AND variation (e.g. standard deviation) or associated estimates of uncertainty (e.g. confidence intervals)
☐	☒	For null hypothesis testing, the test statistic (e.g. *F*, *t*, *r*) with confidence intervals, effect sizes, degrees of freedom and *P* value noted<br>*Give P values as exact values whenever suitable.*
☒	☐	For Bayesian analysis, information on the choice of priors and Markov chain Monte Carlo settings
☒	☐	For hierarchical and complex designs, identification of the appropriate level for tests and full reporting of outcomes
☒	☐	Estimates of effect sizes (e.g. Cohen's *d*, Pearson's *r*), indicating how they were calculated

Our web collection on statistics for biologists contains articles on many of the points above.

Software and code

Policy information about availability of computer code

Data collection	the Tucker-Davis Technology system III (Tucker-Davies Technologies, TDT, Gainesville, FL, USA) for ABR testing; Nikon A1R for imaging; Olympus microscope (BX51WI), Axopatch1500B amplifier and pCLAMP10 software (Molecular Devices) for whole-cell patch-clamp recording; a field emission scanning electron microscope (JSM-7800F prime, JEOL Ltd.) for SEM imaging.
Data analysis	GraphPad Prism 6.07 and ImageJ 1.51h

For manuscripts utilizing custom algorithms or software that are central to the research but not yet described in published literature, software must be made available to editors/reviewers. We strongly encourage code deposition in a community repository (e.g. GitHub). See the Nature Research guidelines for submitting code & software for further information.

Data

Policy information about availability of data

All manuscripts must include a data availability statement. This statement should provide the following information, where applicable:

- Accession codes, unique identifiers, or web links for publicly available datasets
- A list of figures that have associated raw data
- A description of any restrictions on data availability

The source data underlying Figs. 1-5 and Supplementary Figs. 1-3, 5 and 6 are provided in the Source Data file. All data are available upon reasonable request.

Field-specific reporting

Please select the one below that is the best fit for your research. If you are not sure, read the appropriate sections before making your selection.

☒ Life sciences ☐ Behavioural & social sciences ☐ Ecological, evolutionary & environmental sciences

For a reference copy of the document with all sections, see nature.com/documents/nr-reporting-summary-flat.pdf

Life sciences study design

All studies must disclose on these points even when the disclosure is negative.

Sample size	No effect size was predetermined. For experiments in mice, all the biological experiments were performed in two replicated or more. For statistical comparison, three replicated or more biological experiments were performed. Three tissues from two human were used.
Data exclusions	Specimens were excluded if cochlear morphology was not retained during the culture. Any damaged cochlea during the surgery were excluded from subsequent studies.
Replication	For experiments in mice, certain data points were replicated and we found similar results with no significant difference in terms of numbers or values.
Randomization	The injection groups were filled by randomly selecting from the same pool of animals for in vivo experiments.
Blinding	All experimental procedures and quantification of results, including cultures, injections, imaging, auditory brainstem response, and tissue histological analysis were done by three independent researchers.

Reporting for specific materials, systems and methods

We require information from authors about some types of materials, experimental systems and methods used in many studies. Here, indicate whether each material, system or method listed is relevant to your study. If you are not sure if a list item applies to your research, read the appropriate section before selecting a response.

Materials & experimental systems

n/a	Involved in the study	
☐	☒	Antibodies
☐	☒	Eukaryotic cell lines
☒	☐	Palaeontology
☐	☒	Animals and other organisms
☐	☒	Human research participants
☒	☐	Clinical data

Methods

n/a	Involved in the study	
☒	☐	ChIP-seq
☒	☐	Flow cytometry
☒	☐	MRI-based neuroimaging

Antibodies

Antibodies used	The following antibodies were used: Myo7a (#25-6790, Proteus Biosciences), Sox2 (#sc-17320, Santa Cruz Biotechnology), NeuN (#12943S, Cell Signaling Technology), Flag (#F3165, Sigma Aldrich), Donkey anti-Mouse IgG (H+L) Highly Cross-Adsorbed Secondary Antibody, Alexa Fluor 488 (# A-21202, ThermoFisher Scientific), Donkey anti-Rabbit IgG (H+L) Highly Cross-Adsorbed Secondary Antibody, Alexa Fluor 647 (# A-31573, ThermoFisher Scientific), Donkey anti-Goat IgG (H+L) Cross-Adsorbed Secondary Antibody, Alexa Fluor 568 (# A-11057, ThermoFisher Scientific), Donkey anti-Rabbit IgG (H+L) Highly Cross-Adsorbed Secondary Antibody, Alexa Fluor 568 (# A-10042, ThermoFisher Scientific), atto 488 phalloidin (# AD488-82, atto-tech).
Validation	All the antibodies are validated by the manufacturers.

Eukaryotic cell lines

Policy information about cell lines

Cell line source(s)	The HEK293T were purchased from the American Type Culture Collection (Manassas, VA, USA).
Authentication	The HEK293T cell line was not authenticated.
Mycoplasma contamination	The HEK293T cell line has been tested for mycoplasma contamination routinely.
Commonly misidentified lines (See ICLAC register)	*Name any commonly misidentified cell lines used in the study and provide a rationale for their use.*

Animals and other organisms

Policy information about studies involving animals; ARRIVE guidelines recommended for reporting animal research

Laboratory animals	C57BL/6 mice of both sexes in an estimated 50:50 ratio were used in this study. P0-3, P10, P14 and P30 mice were used.
Wild animals	The study did not use wild animals.
Field-collected samples	The study did not involve samples collected from the field.
Ethics oversight	All animal experiments were approved by the Institutional Animal Care and Use Committee of ShanghaiTech University and Southeast University, China.

Note that full information on the approval of the study protocol must also be provided in the manuscript.

Human research participants

Policy information about studies involving human research participants

Population characteristics	The vestibular sensory organs were collected from two patients during the routine tumor resection surgery through translabyrinthine approach, a 50 years old female and a 49 years old male, who were diagnosed with acoustic neuroma and accompanied by severe sensorineural hearing loss.
Recruitment	no selection bias
Ethics oversight	This project was approved by the Research Ethics Committee of the Institution of Eye and ENT Hospital of Fudan University.

Note that full information on the approval of the study protocol must also be provided in the manuscript.
